# Electrically driven optical metamaterials

**DOI:** 10.1038/ncomms12017

**Published:** 2016-06-22

**Authors:** Quynh Le-Van, Xavier Le Roux, Abdelhanin Aassime, Aloyse Degiron

**Affiliations:** 1 Centre de Nanosciences et de Nanotechnologies, CNRS, Univ. Paris-Sud, Université Paris-Saclay, C2N–Orsay, 91405 Orsay cedex, France

## Abstract

The advent of metamaterials more than 15 years ago has offered extraordinary new ways of manipulating electromagnetic waves. Yet, progress in this field has been unequal across the electromagnetic spectrum, especially when it comes to finding applications for such artificial media. Optical metamaterials, in particular, are less compatible with active functionalities than their counterparts developed at lower frequencies. One crucial roadblock in the path to devices is the fact that active optical metamaterials are so far controlled by light rather than electricity, preventing them from being integrated in larger electronic systems. Here we introduce electroluminescent metamaterials based on metal nano-inclusions hybridized with colloidal quantum dots. We show that each of these miniature blocks can be individually tuned to exhibit independent optoelectronic properties (both in terms of electrical characteristics, polarization, colour and brightness), illustrate their capabilities by weaving complex light-emitting surfaces and finally discuss their potential for displays and sensors.

## Abstract (web summary)

Active metamaterials are largely controlled by light, preventing integration in electronic systems. Here, the authors introduce electroluminescent metamaterials based on metal nano-inclusions hybridized with colloidal quantum dots and use this approach to weave intricate light-emitting surfaces.

## Graphical abstract

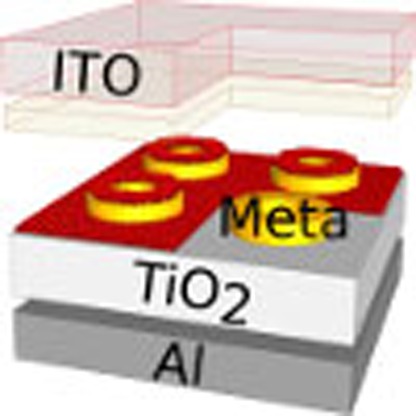

Although most of the signature effects associated with metamaterials^1^,^2^ (negative index, perfect absorption and cloaking) have been successfully demonstrated at near-infrared and visible wavelengths^3^,^4^,^5^,^6^, there are still doubts about the exact potential of artificial optical media for real applications. Part of the problem is that optical metamaterials are often lossy^7^; moreover, they are notoriously difficult to fabricate, because their unit cells must be smaller than the wavelength and thus should not exceed a few hundreds of nanometres at most. As a result, many recent developments have occurred with either stacks of continuous thin films^8^ or with two-dimensional arrays of subwavelength inclusions^9^ (metasurfaces) that, although fascinating in their own right, bear little resemblance with radiofrequency, microwave and terahertz metamaterials. Challenges are even more formidable for one who wishes to build active components that are not controlled or pumped by light but by an electrical signal. At lower frequencies, electrically driven structures can be implemented by incorporating lumped electronic elements in the metamaterial architecture^10^ or by forming Schottky contacts between metallic unit cells and a semiconducting substrate^11^. In the optical regime, however, lumped elements are simply too big compared with the wavelength and alternative implementations must take into account that the electrodes needed to inject an electrical signal occupies a much larger footprint than the metamaterial inner structure and may thus perturb its properties or prevent light from properly interacting with it.

Thus, as is the case with passive components, the future of active optical metamaterials must be envisioned as different from the developments already achieved at terahertz and microwave frequencies. In this study, we introduce electrically driven metamaterials that offers opportunities for solid-state lighting and sensing. We demonstrate the concepts with infrared LEDs exhibiting a form of discrete artificial electroluminescence (EL) and then discuss the importance of our findings for other optoelectronic devices.

## Results

### Device architecture

**Figure 1:**
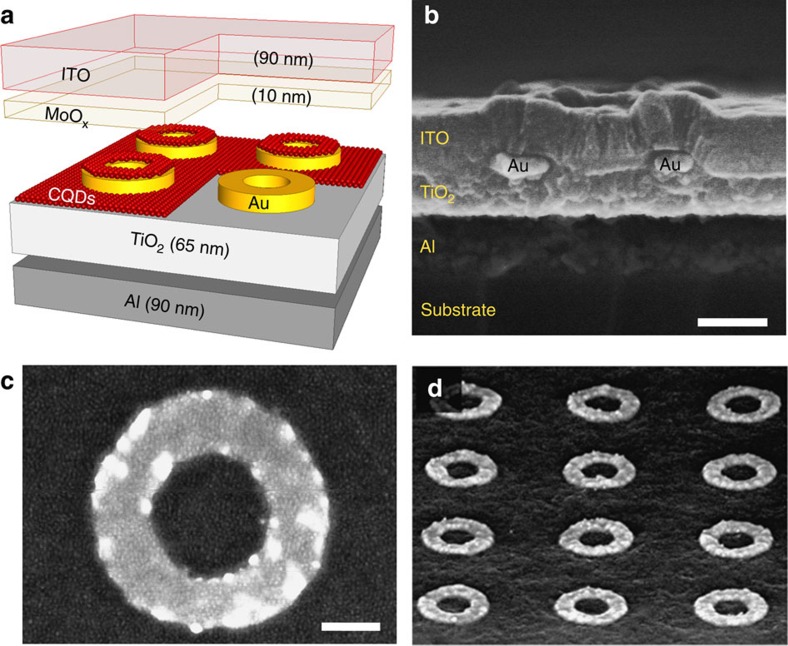
A metamaterial LED. (**a**) Schematic view of the device. (**b**) SEM cross-sectional view of a real structure. The two Au regions correspond to the section of a nanoring. Scale bar, 100 nm. (**c**) An SEM micrograph of a gold nanoring coated with the PbS CQDs. Scale bar, 80 nm. (**d**) A tilted SEM view of the same sample (also coated with CQDs). The period is 600 nm, the inner and outer radii of the rings are 73 and 157 nm, respectively, and the gold thickness is 25 nm.

The all-inorganic architecture of our metamaterials is derived from the quantum dot light-emitting diodes (QLEDs)^12^,^13^,^14^ and photovoltaic cells^15^ developed as cheaper and more versatile alternatives to epitaxial optoelectronic components^16^. As depicted in Fig. 1a and in the cross-sectional view of Fig. 1b, it consists of an Al cathode, a mesoporous film of TiO_2_ for injecting the electrons, a 25-nm-thick gold nanoring array, a 15-nm-thick film of self-assembled PbS colloidal quantum dots (CQDs) purchased from Evident Technologies, a thin layer of MoO_*x*_ for injecting the holes and a transparent indium tin oxide (ITO) anode. All the fabrication details are given in the Methods section. The ring geometry has been chosen because its optical properties do not depend on the in-plane polarization and all the Au nanoparticle arrays considered in this study have a subwavelength period. Figure 1c shows a scanning electron micrograph of a gold nanoring after spin-casting the CQDs on the sample (before deposition of the MoO_*x*_ hole transfer layer and the ITO electrode). The dots, which form a granular assembly of white 5-nm-wide spots separated by a darker corona (trioctylphosphine oxide ligands), clearly cover both the nanoring and the underlying TiO_2_ surface.

**Figure 2:**
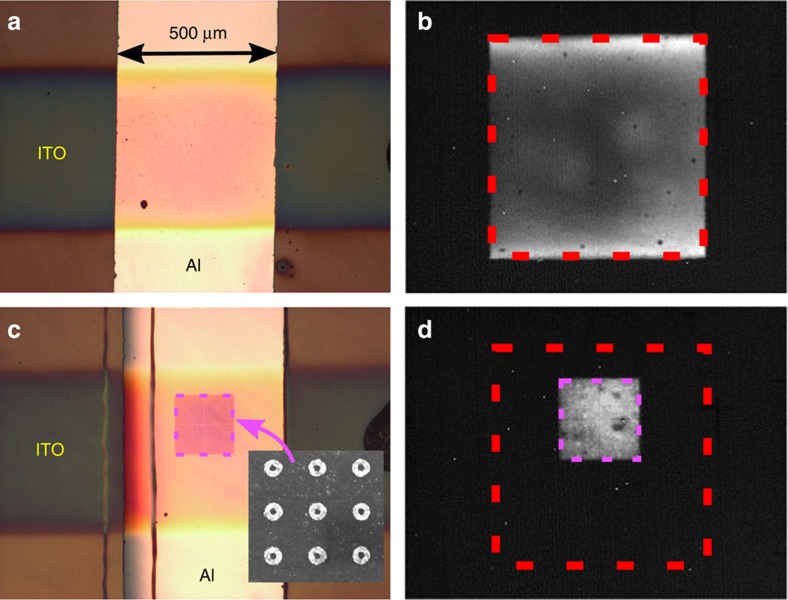
Images of fabricated devices and infrared EL. (**a**) Optical image of a reference sample. The junction is defined by the overlap of the ITO and Al electrode. (**b**) Infrared EL of the same junction for a positive bias voltage of 10 V (because the scale is different from **a**, we highlight the boundaries of the 500 × 500 μm^2^ junction with a red dashed square). (**c**) Optical image of a metamaterial LED. The gold nanoparticle array appears as a purple square (highlighted with a magenta dashed stroke). (**d**) Infrared EL of the metamaterial LED for a positive bias voltage of 4 V (same scale as **b**).

The Al cathode and ITO anode have a width of 500 μm and are perpendicular to each other, so that the only region that can be pumped electrically is the square area where they overlap. In practice, a matrix of 12 independent LEDs is fabricated on each sample. We define a different gold nanoparticle array for ten of these structures and leave the two remaining diodes without metal structuration to use as reference devices. In these reference QLEDs, the light-emitting area appears as a light orange-shaded square corresponding to the region where the ITO and Al electrodes overlap (Fig. 2a). When a positive bias voltage is applied, infrared light is emitted from this square zone in a uniform way (Fig. 2b) and we have verified that no light occurs for negative biases.

### Properties of the metamaterial LEDs

We now turn our attention to the operation of a metamaterial LED, illustrated in Fig. 2c,d. In this example, the nanoring array has a period 450 nm, inner ring radius 30 nm and outer ring radius 88 nm. The surface occupied by this array is limited to 200 × 200 μm^2^; thus, it is smaller than the total size of the LED (500 × 500 μm^2^). This particularity can be seen on the picture of Fig. 2c where the nanoring array appears as a purple square within the larger orange-shaded junction. When a positive voltage is applied to the electrodes, light emission only occurs above the region occupied by the nanoring array rather than above the full surface occupied by the LED (Fig. 2d).

**Figure 3:**
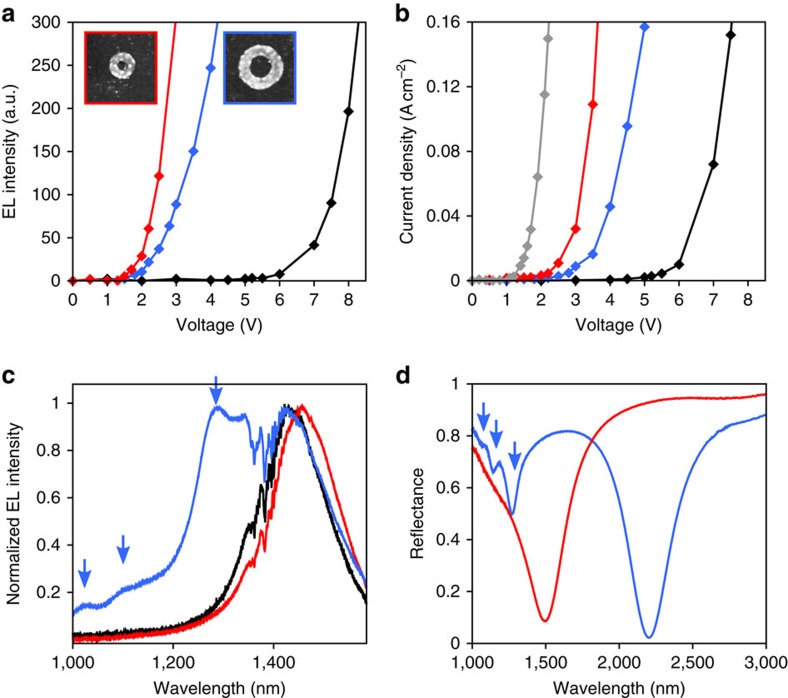
Properties of the metamaterial LEDs. (**a**) *L*–*V* curves for the reference QLED (black), the metamaterial LED with small rings of Fig. 2 (red curve) and the metamaterial LED of Fig. 1 with large rings and period (blue curve). This colour code is used in the other panels of the figure. (**b**) *J*–*V* curves for the same devices. Also shown is the *J*–*V* curve of a QLED where the nanoparticle array has been replaced by a continuous gold film with the same thickness (in grey). (**c**) EL spectrum of the three devices, with the plasmonic resonances of the blue curve highlighted with arrows. The noise in the 1,345–1,480 nm window is due to the absorption lines of the atmosphere. The small peak at 1,345 nm, best seen on the blue curve, is also an artefact caused by these absorption lines. (**d**) FTIR reflectance spectra of the metamaterial LEDs. All these properties are stable even after numerous electrical pumping cycles. Moreover, the metamaterial LEDs are capable of emitting infrared light over several hours of consecutive pumping (Supplementary Fig. 4).

To gain quantitative insight, Fig. 3a reports the integrated EL intensity of the devices as a function of the applied voltage. These light–voltage (*L*–*V*) curves are normalized by the effective light-emitting area of each structure to allow for quantitative comparisons between the reference QLED that produces light from the entire junction and the metamaterial LED that emits light only above the nanoring array. In the case of the reference QLED (black curve), EL becomes significant above a threshold voltage of 5.5 V. This high turn-on bias can be explained by the fact that we do not perform any step (such as replacing the long trioctylphosphine oxide ligands capping the nanocrystals with shorter ones) to reduce the energy barriers between the CQDs and adjacent layers. If we now consider the metamaterial LED, we observe a dramatic change of the *L*–*V* characteristics, with a turn-on voltage as low as 1.3 V. It is accompanied by a much steeper *L*–*V* curve, so that the EL, at any given voltage, is enhanced by several orders of magnitude with respect to the conventional QLED. Importantly, we have verified that these properties do not change with the size of the nanoring array (Supplementary Figs 1–2). On the other hand, the *L*–*V* characteristics can be tuned with the geometry of the Au nanoparticle array: for example, we show in the same panel that a metamaterial LED with larger rings and larger period (the geometrical parameters are those of Fig. 1) exhibits a slightly higher turn-on voltage (blue curve). The *L*–*V* curves of the devices are strongly correlated with their current density–voltage (*J*–*V*) behaviour, as evidenced in Fig. 3b and it should be noted that the *L*–*V* and *J*–*V* characteristics exhibit a small hysteresis (not shown in Fig. 3 for clarity but plotted in Supplementary Fig. 3). We attribute this behaviour to the presence of TiO_2_ in our LEDs—a material well-known for its memristive properties^17^.

For the sake of completeness, we have also performed two additional control experiments. First, we have repeated the measurements for a device without CQD and verified that no light was emitted in this case; second, we have measured the properties of a structure in which the Au nanoring array has been replaced by a continuous gold film. Its *J*–*V* characteristic, plotted in grey in Fig. 3b, has an even better conductivity than the metamaterial LEDs. This result indicates that the presence of gold in the stack induces a path of least resistance where the current preferentially flows. However, this mechanism alone does not explain the operation of the metamaterial LEDs, because the device with the continuous metal film does not emit light even at high voltages. In addition, the metamaterial LED with the highest brightness and conductivity (small rings and period 450 nm, red curves of Fig. 3a,b) is the one where the fraction occupied by the gold patterns is the smallest (11% of the total ring array, as opposed to 17% for the metamaterial LED with larger rings and larger period characterized in the same panels). Thus, our results cannot be explained solely because the presence of Au reduces the resistivity of the junction.

To identify the dominant mechanism governing the metamaterial LEDs, we plot in Fig. 3c the EL spectra of the devices. The reference QLED is characterized by a broad spectral distribution around 1,450 nm that reflects the inhomogeneous broadening of the dots, the EL spectrum of the metamaterial LED with small rings is slightly shifted to longer wavelengths (red curve), whereas the metamaterial LED with large rings exhibits much more complex spectral features such as the apparition of three new peaks at smaller wavelengths (blue curve). It is noteworthy that all spectra have been normalized to ease the comparison. In reality, their absolute intensities at a given bias voltage are very different, as they follow the *L*–*V* curves presented in Fig. 3a.

The fact that the metamaterial LEDs exhibit resonances that depend on the geometry of the gold nanostructures is indicative of the excitation of localized surface plasmons. To confirm this hypothesis, we have measured the reflectance spectrum of the devices by Fourier transform infrared (FTIR) spectroscopy. The structures are illuminated from the ITO side with a Cassegrain objective. Owing to the presence of the Al electrodes on the back side of the sample, most of the incoming light is either reflected back towards the objective and the FTIR spectrometer or, if a surface plasmon resonance is excited, scattered and absorbed, producing a distinctive minimum in the reflection spectrum. The experimental curves are plotted in Fig. 3d. Similar to the EL data of Fig. 3c, the FTIR spectrum of the metamaterial LED with small rings exhibits a single broad resonance at 1,500 nm (red curve) that corresponds to the fundamental plasmon mode of the nanorings (higher-order resonances are also supported at wavelengths smaller than those plotted in Fig. 3d). The structure with larger rings has essentially the same reflectance properties, except that the scaling of the geometrical dimensions shifts the plasmonic resonances to larger wavelengths (blue curve of Fig. 3d). In this case, the fundamental plasmon resonance occurs at 2,200 nm, which is outside the spectral range of emission of the LED, but one can see that three higher-order plasmon resonances are excited at 1,280, 1,130 and 1,060 nm, respectively (blue arrows). A numerical description of these modes has already be given elsewhere^18^; thus, we only note here that they are observed at the same wavelengths as the three EL maxima of Fig. 3c.

Thus, the FTIR resonances of Fig. 3d show an excellent correlation with the EL peaks of Fig. 3c, indicating that the plasmonic resonances play a central role in the operation of the metamaterial LEDs. To further substantiate this claim, we have also measured the photoluminescence spectra of the three devices and observed the same general features (Supplementary Fig. 5). It should be noted that the blue curve of Fig. 3c exhibits a fourth peak at 1,450 nm that does not appear in the FTIR spectrum of Fig. 3d. This peak can be explained by the fact that the plasmonic resonances in Fig. 3c are convoluted with the EL spectrum of the CQDs in the absence of the nanorings and we know from the EL spectra of the reference QLED that the latter exhibits a maximum at this wavelength (black curve of Fig. 3c). The 1,450-nm-peak also contributes to the EL spectra of the device with small rings (red curve of Fig. 3c) but it is not visible, because it overlaps almost totally with the fundamental plasmon resonance at 1,500 nm. These features are consistent with the fact that the metamaterial LED with small rings is the brightest device (red curve of Fig. 3a): the surface plasmon resonance, occurring at 1,500 nm, is almost exactly matched with the maximum of EL of the PbS CQDs. In contrast, the plasmonic boost provided by the larger rings of the other metamaterial LED occurs at wavelengths where the PbS CQDs emit less light, resulting in a lower absolute EL intensity.

### Role of the plasmonic resonances

There are several contexts in which the light emission of quantum emitters is boosted by the presence of plasmonic nanostructures and, more particularly, in fluorescence experiments and calculations^19^,^20^,^21^,^22^. The various enhancements reported in these studies are in part due to the fact that surface plasmons are characterized by intense electromagnetic fields that considerably increase the number of radiative decay channels for the dots and therefore improve their quantum yield (Purcell effect). Several QLEDs have been demonstrated with charge transfer layers containing plasmonic nanoparticles that enhance the emissivity^23^,^24^,^25^ with the Purcell effect. In these studies, the nanoparticles were not in the immediate vicinity of the active CQD layer, the EL was boosted by a few times and the electrical characteristics and spectra did not change noticeably. Here, in contrast, the dots are directly coated on the gold ring arrays and thus we are in a different regime where their interaction with the surface plasmon resonances is much stronger and where at least one additional effect contributes to the light emission mechanism (the fact that the presence of Au induces a path of least resistance, as discussed previously). Further studies are needed to fully understand why this new configuration does not only enhance the brightness of the LEDs by orders of magnitude at any given voltage, but also dramatically improves the charge injection, conductivity and threshold voltage (Fig. 3a,b). It is possible that in our case, the plasmonic boost is not solely mediated by the electromagnetic field, but also by non-radiative energy transfers and cooperative effects, because the CQDs form a compact carpet directly on top of the metal nanoparticles. Importantly, we found that the properties of Figs 2 and 3 disappear if the CQD film becomes thicker than ∼30 nm. We interpret this behaviour by the fact that thicker CQD films are mainly composed of dots that are too far from the metal inclusions, creating a barrier that absorbs light and prevents increased charge injection in the few monolayers of PbS nanocrystals placed in the direct vicinity of the Au inclusions.

## Discussion

**Figure 4:**
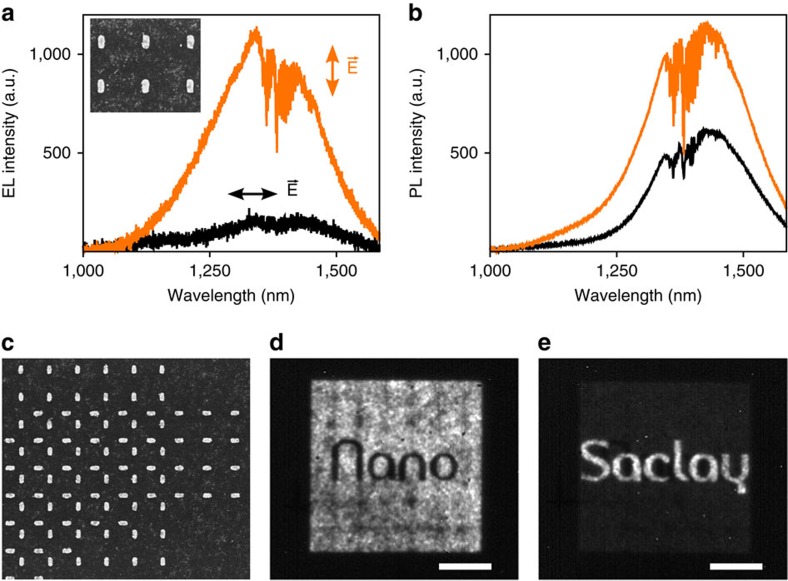
A true form of artificial EL. (**a**) EL properties of a metamaterial LED operating with an array of gold nanorods with length 150 nm, width 70 nm and period 500 nm. Orange (black) curve: spectrum of the light emitted with a polarization parallel (perpendicular) to the long axis of the nanorods. Inset: SEM view of the array. (**b**) Photoluminescence spectra of the device for the same two orthogonal polarizations. (**c**) A detail of the ‘NanoSaclay' LED featuring two sets of nanorods with the same dimensions and period as in **a** and **b**. (**d**,**e**) Infrared images of the ‘NanoSaclay' LED, as seen through a linear polarizer successively aligned with the vertical set of nanorods and the horizontal one. Scale bars, 30 μm.

We now explain why a Au nanoparticle array coated with CQDs qualifies as a genuine artificial active medium. To this aim, consider the metamaterial LED of Fig. 4a operating with an array of lithographed gold nanorods, all the other parameters being identical to those of Figs 1, 2, 3. Owing to their geometry, the plasmonic resonances can only be excited and re-emit light with an electric field parallel to their long axis. This polarization dependence dominates the response of the metamaterial LED, as shown in Fig. 4a where most of the light emitted by the structure is polarized along the long axis of the rods. This result is all the more remarkable that the nanorods occupy a small fraction of the total surface of the array (4.2%), implying that the CQDs that are not coupled to them are not active. For comparison, we show in Fig. 4b the photoluminescence spectra of the device for the two same orthogonal polarizations. In this experiment, a 632-nm laser beam is focused on the sample with a × 50 objective that excites all the dots under the focus spot (that is, those that are coupled to the rods and those that are not). The resulting spectra are also sensitive to the polarization but in a strikingly less remarkable way than in Fig. 4a, because the vast majority of the dots are not coupled to the nanorods and emit unpolarized light.

These results demonstrate that the metamaterial LEDs exhibit a form of structural EL with discrete, subwavelength elements. Not only can we tailor the electrical and optical properties of the structure, as seen before, but we can also conclude that each gold inclusion and the dots in its immediate proximity form an active pixel of subwavelength dimensions that can be used to weave complex light-emitting surfaces. As an illustration, we have fabricated a metamaterial LED displaying different words depending on the polarization through which we look at the structure: the word ‘Nano,' appearing in black over a white background, and the word ‘Saclay,' appearing in white over a black background. The structure contains two arrays, one made of vertically oriented nanorods with voids corresponding to the word ‘Nano' and the second with horizontally oriented nanorods shaping the word ‘Saclay'. A detail of the two intertwining arrays appears in Fig. 4c, whereas the light emission of the fabricated device is shown in Fig. 4d,e for the two orthogonal polarizations. Clearly, each word appears distinctively for one polarization only, demonstrating that the nanorods coupled with their surrounding dots act as discrete pixels as intended.

It should be noted that in all the cases discussed in this study, the pixels are separated by a subwavelength distance so that it is not possible to distinguish them when one observes the resulting light emission in the far field (Figs 2d and 4d,e). In this regime, a Au nanoparticle array coupled with CQDs operates as an effective active medium (that is, as a metamaterial), because the light emitted from the structures seems to come from a uniform active layer, whereas in fact it is emitted by a set discrete elements. This approach makes it possible to emulate a wide variety of effective behaviours, as we have seen that the light-emitting pixels can be individually tuned to control all the aspects of the light emission at the nanoscale (polarization, spectral properties, electrical injection and brightness; see also Supplementary Fig. 6 for an example where the metamaterial approach is used to emulate several materials with different bandgaps within the same LED).

Among the possibilities offered by this approach, it should be possible to enable a phase coherence between the different plasmonic resonances^26^ and thus achieve a collective behaviour to shape complex beams or generate holographic images. The fact that the plasmonic resonances enhance the emission is also promising for developing optical sources with a high modulation rate, as suggested recently^27^. Beyond light-emitting devices, our findings may also be applicable for diodes operating as photodetectors. In particular, it would be interesting to use the same metamaterial approach to build sensors with nanoscale pixels and exploit the plasmonic enhancement to obtain ultra-short responses—a much needed property at infrared wavelengths where ultra-fast charge-coupled device cameras do not operate. Apart from these immediate perspectives, it is worth noting that the metamaterials presented in this study combine two types of artificial atoms—the metallic unit cells of plasmonic arrays on the one hand and quantum dots on the other. Thus, our research shows the enormous synergy of combining different types of artificial matter and suggests that many other opportunities will arise by taking a unified view of the various artificial media developed by the physics, chemistry and engineering communities.

## Methods

### Fabrication

The fabrication starts with 2' wafers of borosilicate glass manufactured by Plan Optik AG that are cut into 1 cm × 1 cm squares with a dicing saw. The resulting substrates are thoroughly cleaned in an ultrasonic bath of acetone, then rinsed in isopropanol, deionized water and dehydrated in an oven at 96 °C. We do not need a more elaborated cleaning procedure, because the 2' wafers are already thoroughly pre-cleaned before shipping.

Four Al cathodes are patterned on each substrate by electron beam evaporation of 80 nm of Al through a shadow mask. The mask is such that the electrodes have a width of 500 μm, a length of 1 cm (corresponding to the length of the substrate) and are separated by 2 mm. A thin Kapton adhesive is then applied on one side of the sample so as to protect one end of the Al electrodes from the coating of the next layer—a 65-nm-thick mesoporous film of anatase TiO_2_ obtained by spin-casting a commercial solution of TiO_2_ nanoparticles (Ti-Nanoxide HT-L/SC from Solaronix) at 5,000 r.p.m. for 60 s, followed by a hot-plate bake at 200 °C during 15 min.

The sample is then ready for the fabrication of the gold nanoparticle arrays using electron-beam lithography, electron-beam evaporation of Ti (2 nm) and Au (25 nm), and lift-off. The resist used for this step is a solution of ZEP520A diluted in anisole with a dilution ratio of 2, on top of which we deposit a thin conductive film of Espacer 300Z to evacuate the charges that would otherwise accumulate during the lithography step. The nanoparticle arrays are written with a Nanobeam NB4 system with an acceleration voltage of 80 kV, a current of 2 nA and a dose of 3 C m^−2^. A manual alignment procedure is used to write the different nanoparticle arrays precisely at the location of the future LEDs (corresponding to the overlap of the Al cathodes and ITO anodes, see main text and Fig. 2 for more info). Each completed sample has 12 LEDs on it, corresponding to the overlap of 4 Al cathodes and 3 ITO anodes; we typically define a nanoparticle array for 10 of these LEDs and let 2 of them without any metal pattern to serve as reference devices.

The next step after the fabrication of the nanoparticle arrays is the deposition of the PbS CQD film. This step is performed with a commercial solution of PbS CQDs in toluene from Evident technologies with a concentration of 26 mg ml^−1^ of CQDs. The radius of the CQDs is 5.3 nm and they are surrounded by 2 nm of trioctylphosphine oxide ligands. This solution is spin coated on the sample (again, we protect one end of the Al electrodes with a thin adhesive) in a fume hood under ambient atmosphere and without any chemical postprocessing (such as ligand exchange) other than a hot-plate bake at 150 °C for 3 min.

The last two layers are deposited with a Denton sputtering system. The first of these layers is a 10-nm-thick film of MoO_*x*_ deposited with reactive RF magnetron sputtering of a 3-inch-wide and 0.125-inch-thick Mo target (99.98%, Kurt J. Lesker) in 15% O_2_–85% Ar at a pressure of 10 μbar and a rate of 0.2 Å s^−1^. The ITO electrodes are deposited in RF magnetron mode with a 3-inch-wide and 0.125-inch-thick ITO target (99.99%, Kurt J. Lesker) through the same shadow mask that was already used to pattern the Al cathodes, except that the mask is rotated 90°. The anodes are actually made of a bilayer of two ITO coatings deposited in an inert argon environment at a pressure of 8 μbar but different sputtering powers. The first layer is 10 nm thick; it is deposited with a low power density (0.65 W cm^−2^) at a rate 0.5 Å s^−1^, to have a polycrystalline coating with small grains designed to maximize adherence to the stack. The second layer is a 80-nm-thick polycrystalline coating with larger grains, having more internal stress but a much higher conductivity.

It should be noted that the sample shown in Fig. 1 of the main article has been made on a Si substrate covered by a 500-nm-thick layer of silica rather than a glass substrate so that we could cleave it and have a nice cross-sectional view of the stack. Before cleaving this sample, we have verified that it was working, and that it exhibited the same characteristics as our samples made on glass.

### Material and morphological characterization of the samples

All samples were monitored during and after the fabrication process. In particular, we used XPS to confirm the chemical composition of key layers. Atomic force microscopy in tapping mode was used to characterize the roughness of the mesoporous TiO_2_ layer (see Supplementary Fig. 7 and roughness information given in the caption). High-resolution scanning electron microscopy (SEM) was also used extensively to inspect the quality of the samples. As discussed in Fig. 1 of the main text, it was with an SEM that we were able to determine how the CQD self-assemble on the TiO_2_ surface patterned with gold inclusions.

### Electrical and optical measurements

For all the experiments involving electrical pumping, the LEDs are connected to a Keithley 2636A sourcemeter that can be automatically programmed to perform a range of pumping cycles. Each sample has a matrix of 4 × 3 LEDs. To power one of these LEDs, we connect the Keithley sourcemeter to the corresponding Al cathode and ITO anode with tungsten tips controlled by micropositioner probes. In the case of the ITO anodes, the tungsten tip is landed on a pad of dried silver paint applied on top of the ITO. Such Ag pads prevent the tip from perforating the ITO electrodes and are applied directly next to each junction. A part of an Ag pad is visible on Fig. 2c (black region on the rightmost part of the ITO anode).

### Infrared images of the EL

The sample is placed under a specially prepared BX51 WI Olympus microscope coupled to an InGaAs camera cooled at −10 °C (model Xeva 1M with 320 × 256 pixels from Xenics) (Figs 2b,d and 4d,e). The images of Figs 2b,d and 4d,e are recorded with a × 10 infrared objective with numerical aperture (NA)=0.25, also from Olympus. To obtain the images of Fig. 4d,e, a Glan–Thomson polarizer is inserted inside the microscope and successively aligned along the vertically oriented and horizontally oriented nanorods.

### *L*–*V* and *J*–*V* measurements

The *L*–*V* measurements of Fig. 3a are obtained by recording a series of images with our InGaAs camera (see previous section), whereas incrementally raising the voltage applied on the device. From these images, we integrate the light intensity and normalize this value by the light-emitting area. Such normalization is necessary to allow for comparison between the reference QLEDs (which emit light across the entire zone where the Al and ITO electrodes overlap; Fig. 2b) and the metamaterial LEDs that emit light only above the Au nanoparticle array (Fig. 2d).

The *J*–*V* measurements of Fig. 3b bare recorded together with the *L*–*V* curve by recording the current flowing through the device for each step voltage.

### EL spectra

Again, we place the sample under our Olympus microscope and pump it electrically; only this time we redirect the EL towards an iHR320 spectrometer from Horiba Jobin-Yvon coupled to a nitrogen-cooled InGaAs detector (Symphony II, also from Horiba Jobin-Yvon) (Figs 3c and 4a). In the case of Fig. 4a, a Glan–Thomson polarizer is inserted before the spectrometer, to analyse the collected light. The polarization-dependent spectra of this figure are obtained by rotating the sample (and not the polarizer), to avoid any artefact from the iHR320 spectrometer that also partially polarizes the incoming light.

We typically use either a × 10 objective with NA=0.25 or a × 50 objective with NA=0.55, to record our spectra. Importantly, we have verified that these spectra are not affected by the NA and objective used, as shown in Supplementary Fig. 8 for a metamaterial LED with nanorings with inner radius 84 nm, outer radius 162 nm and period 600 nm.

### FTIR reflectance spectra

These measurements have been performed with a Variant FTIR spectrometer coupled with an optical microscope (Fig. 3d). The structures are illuminated from the ITO side with a Cassegrain microscope with a range of angles between 15° and 30° with respect to the normal direction. The data are normalized by the reflectivity of a gold-coated substrate.

### Data availability

The authors declare that the data supporting the findings of this study are available within the article and its Supplementary Information files.

## Additional information

**How to cite this article**: Le-Van, Q. *et al*. Electrically driven optical metamaterials. *Nat. Commun.* 7:12017 doi: 10.1038/ncomms12017 (2016).

## Supplementary Material

Supplementary Information Supplementary Figures 1-8.
