# Supplementary material for: Electrically driven optical metamaterials
Source: Nat Commun. 2016 Jun 22;7:12017. doi: 10.1038/ncomms12017 (PMC4917961; doi:10.1038/ncomms12017)
Supplement: Supplementary Information — Supplementary Figures 1-8. [file ncomms12017-s1.pdf]

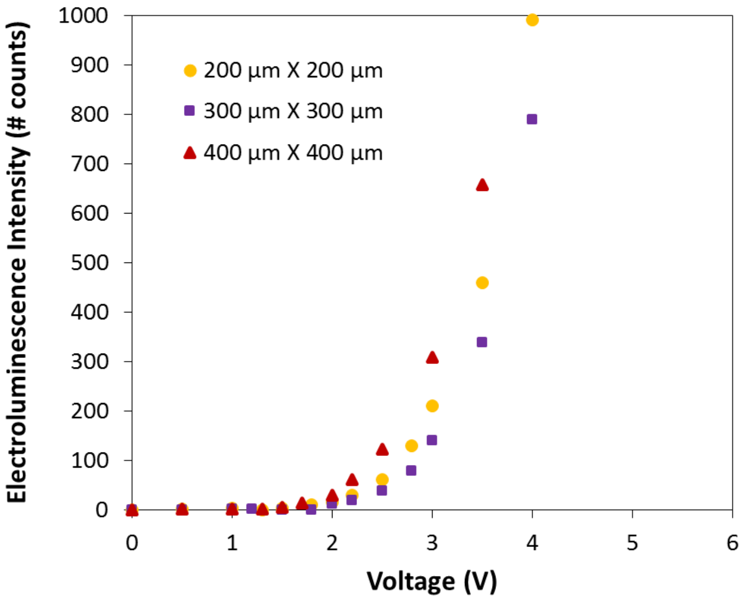

Supplementary Fig. 1. L-V characteristics of a series of metamaterial LEDs with the same parameters as the one characterized on Fig. 3a, red curve (gold nanoring array with period 450 nm, inner ring radius 30 nm and outer ring radius 88 nm), but with ring arrays ranging from 200 x 200 μm^2 to 400 x 400 μm^2.

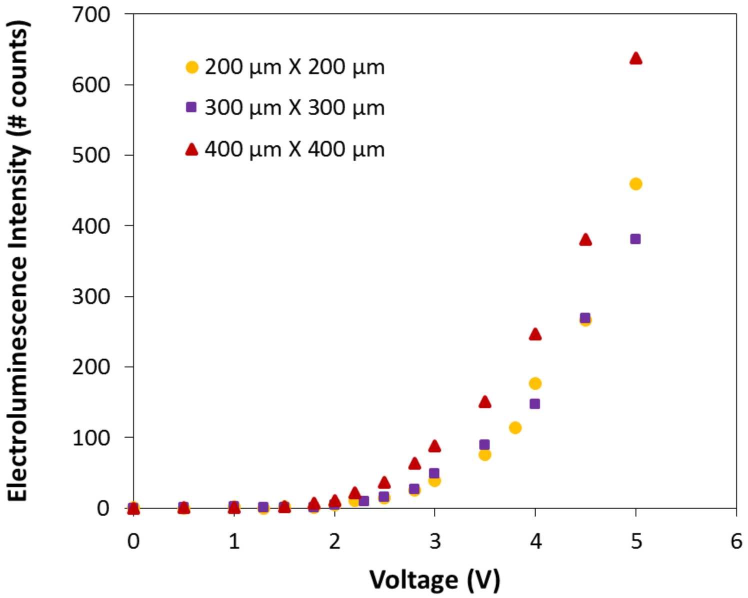

Supplementary Fig. 2. L-V characteristics of a series of metamaterial LEDs with the same parameters as the one characterized on Fig. 3a, blue curve (gold nanoring array with period 600 nm, inner ring radius 73 nm and outer ring radius 157 nm), but with ring arrays ranging from 200 x 200 μm^2 to 400 x 400 μm^2.

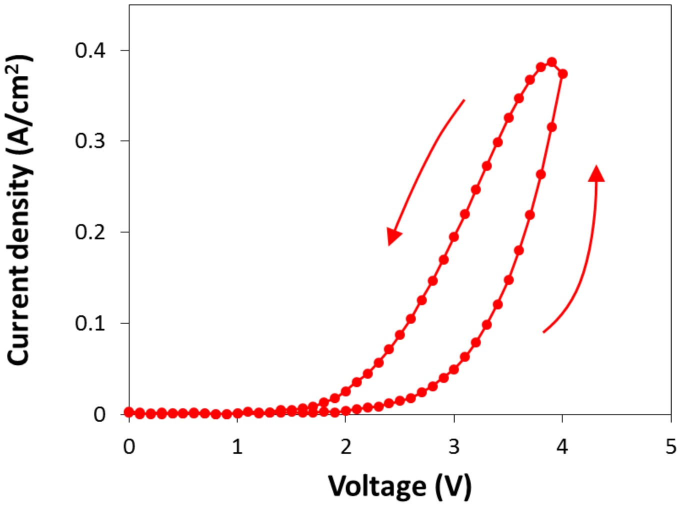

Supplementary Fig. 3 | Typical hysteresis curve of our metamaterial LEDs. Here we show the hysteresis of the metamaterial LED with large rings (inner radius 73 nm, outer radius 157 nm and period 600 nm) discussed in the main text (blue curves of Fig. 3). In the main text (Figs. 3a and 3b), we only show the portion of the curve where we ramp up the voltage for more clarity.

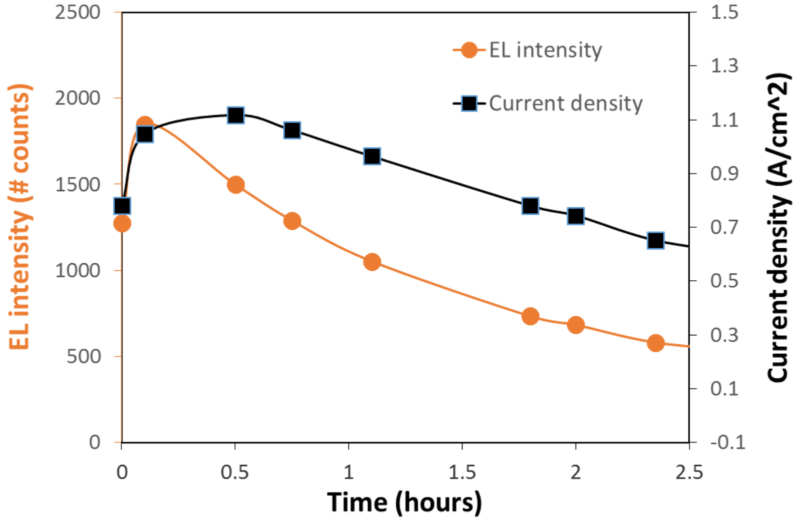

Supplementary Fig. 4 | Evolution of the electroluminescence and current density as a function of time. Here we measure the metamaterial LED with small rings (inner radius 30 nm, outer radius 88 nm and period 450 nm) discussed in the main text (red curves of Fig. 3). The applied bias voltage is 4.5 V. The curve shown here is typical of our metamaterial LEDs although it should be noted that we were sometimes able to obtain structures with no visible decrease of the light intensity after two hours or more of continuous pumping. We attribute these variations to the fact that we fabricate our structures under ambient atmosphere, which is known to induce some variability in terms of the robustness of the devices.

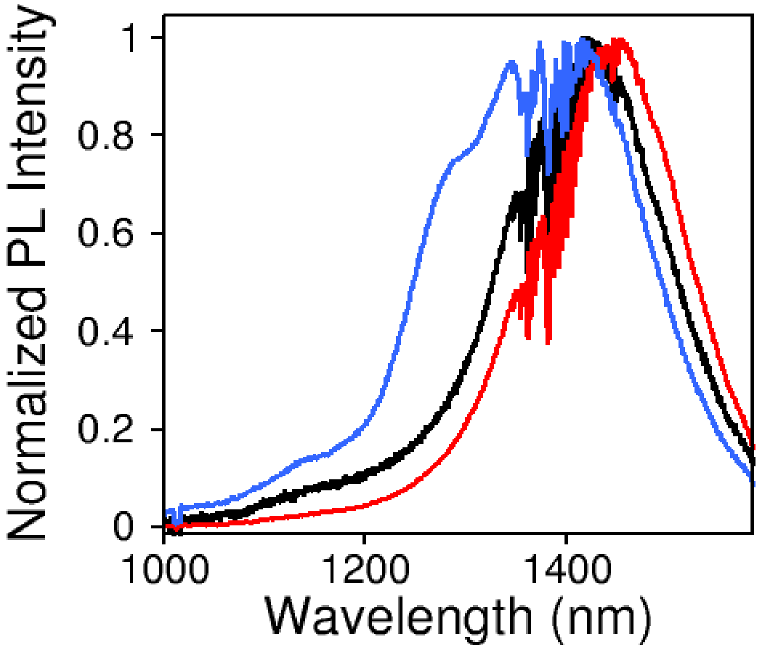

Supplementary Fig. 5 | Photoluminescence (PL) measurements of the devices. Same color code as in the text (black curve: PL of the reference device without rings; red curve: PL of the metamaterial LED with small rings; blue curve: PL of the metamaterial with large rings). The PL of the metamaterial LEDs taken above regions far from the rings (not plotted here) superimposes with the black curve corresponding to the reference device without rings. The sharp notch in the spectra at 1010 nm is an experimental artifact. The spectra have been obtained by pumping the structures with a 632 nm He-Ne laser and have been normalized for better comparison with Fig. 3c of the main text.

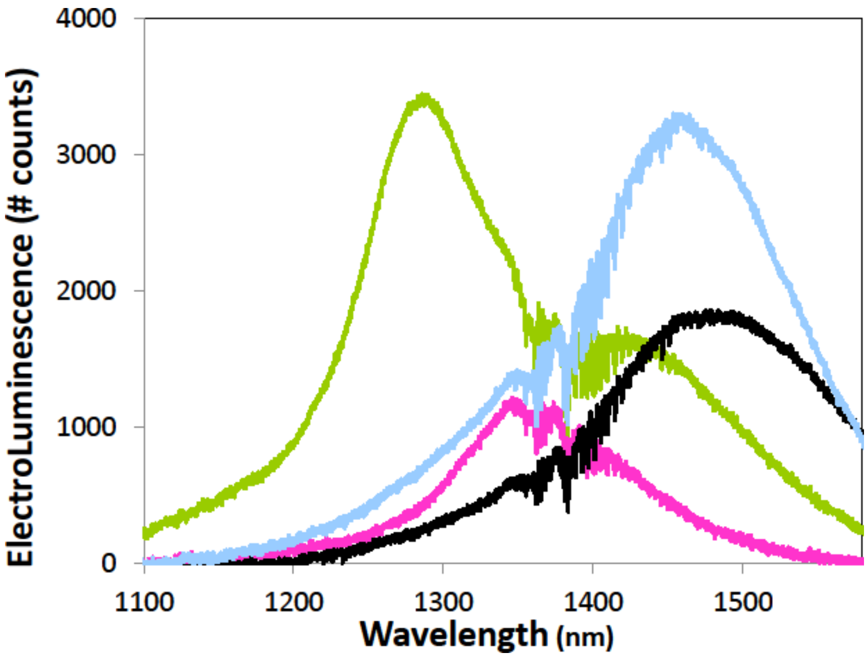

Supplementary Fig. 6 | A metamaterial LED with light-emitting zones of different color. Here we have fabricated a metamaterial LED in which the geometry varies from one zone to another. Green curve: matrix of nanorings with inner radius 73 nm, outer radius 157 nm and period 600 nm. Magenta curve: nanorings with inner radius 84 nm, outer radius 162 nm and period 600 nm. Blue curve: rings with inner radius 30 nm, outer radius 88 nm and period 450 nm. Black curve: rings with inner radius 45 nm, outer radius 92 nm and period 600 nm. Thus, each portion of the same LED emits very different colors with different intensities, as if it were made of different materials with different bandgaps. Note that the green and blue curves correspond to nanoring geometries used in the main text but that the resulting spectra are different. This is because we show here a sample with a TiO_2 thickness reduced to 50 nm, perturbing the plasmonic resonances that are in closer proximity to the Al electrode.

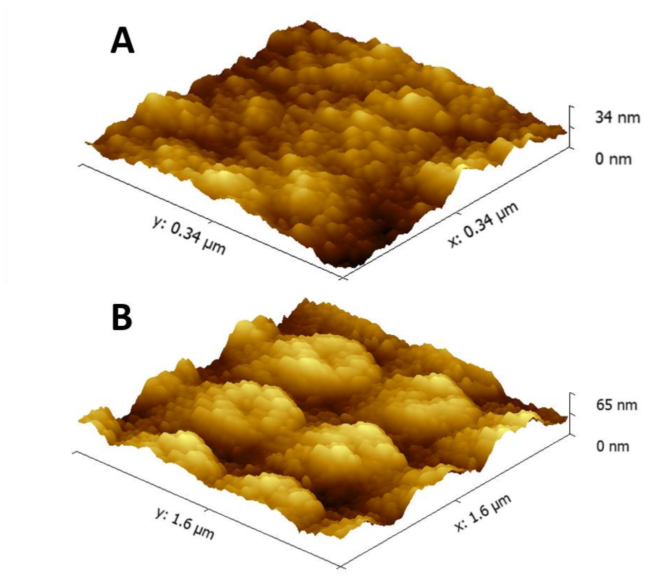

Supplementary Fig. 7 | AFM characterization of the roughness of the TiO_2 layer. (**a**) AFM view of the TiO_2 layer far from any gold metasurface. Mean (arbitrary) altitude: 17 nm, rms roughness 4.6 nm. (**b**) AFM view of the TiO_2 layer with gold nanorings patterned on top of it. These are the same nanorings that appear in Figs. 1c and 1d of the main text but their center is not resolved because of the size of the AFM tip. Note: the scales of the two maps are different.

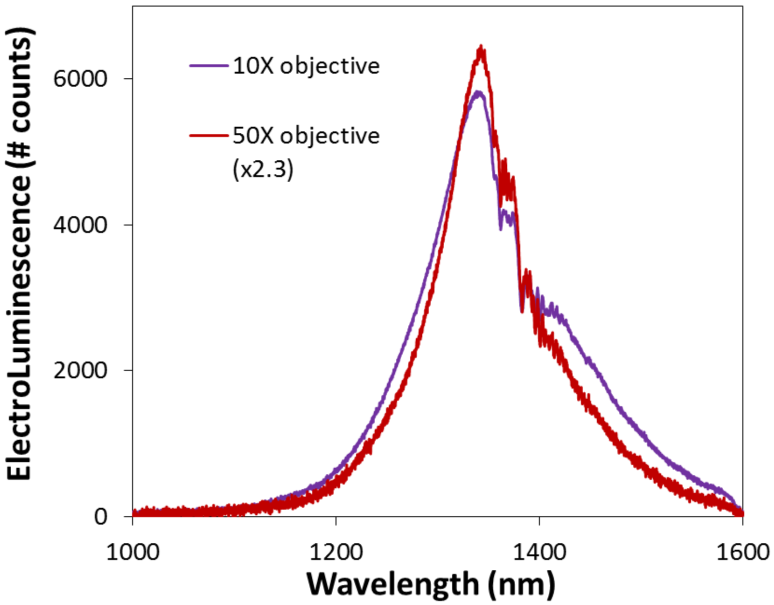

Supplementary Fig. 8 | Control experiment. Electroluminescence spectrum of a metamaterial LED taken with two different objectives. Violet curve: 10X objective, N.A.=0.25. Red curve: 50X objective, N.A.=0.55.
